# Study protocol for a randomized controlled trial comparing the efficacy of a specialist and a generic parenting programme for the treatment of preschool ADHD

**DOI:** 10.1186/1745-6215-15-142

**Published:** 2014-04-25

**Authors:** Donna C McCann, Margaret Thompson, David Daley, Joanne Barton, Cathy Laver-Bradbury, Judy Hutchings, David Coghill, Louise Stanton, Tom Maishman, Liz Dixon, Josh Caddy, Maria Chorozoglou, James Raftery, Edmund Sonuga-Barke

**Affiliations:** 1 Developmental Brain and Behaviour Lab, Psychology, University of Southampton, Southampton SO17 1BJ, UK; 2 Child and Adolescent Mental Health Services, Solent NHS Trust, Orchard Centre, Southampton SO16 4XE, UK; 3 Division of Psychiatry and Applied Psychology, University of Nottingham, Nottingham NG7 2TU, UK; 4 North Staffordshire Combined Healthcare NHS Trust, Stoke-on-Trent ST1 5UK, UK; 5 School of Psychology and Centre for Evidence Based Early Intervention, Bangor University, Bangor LL57 2PX, UK; 6 School of Medicine, University of Dundee, Ninewells Hospital, Dundee DD1 9SY, UK; 7 University of Southampton Clinical Trials Unit, Southampton General Hospital, Southampton SO16 6YD, UK; 8 Wessex Institute for Health, Research and Development, University of Southampton, Southampton Science Park, Southampton SO16 7NS, UK

**Keywords:** ADHD, cost-effectiveness, effectiveness, parenting, parenting programme, parent training

## Abstract

**Background:**

The New Forest Parenting Programme (NFPP) is a home-delivered, evidence-based parenting programme to target symptoms of attention-deficit/hyperactivity disorder (ADHD) in preschool children. It has been adapted for use with ‘hard-to-reach’ or ‘difficult-to-treat’ children. This trial will compare the adapted-NFPP with a generic parenting group-based programme, Incredible Years (IY), which has been recommended for children with preschool-type ADHD symptoms.

**Methods/design:**

This multicentre randomized controlled trial comprises three arms: adapted-NFPP, IY and treatment as usual (TAU). A sample of 329 parents of preschool-aged children with a research diagnosis of ADHD enriched for hard-to-reach and potentially treatment-resistant children will be allocated to the arms in the ratio 3:3:1. Participants in the adapted-NFPP and IY arms receive an induction visit followed by 12 weekly parenting sessions of 1½ hours (adapted-NFPP) or 2½ hours (IY) over 2.5 years. Adapted-NFPP will be delivered as a one-to-one home-based intervention; IY, as a group-based intervention. TAU participants are offered a parenting programme at the end of the study. The primary objective is to test whether the adapted-NFPP produces beneficial effects in terms of core ADHD symptoms. Secondary objectives include examination of the treatment impact on secondary outcomes, a study of cost-effectiveness and examination of the mediating role of treatment-induced changes in parenting behaviour and neuropsychological function. The primary outcome is change in ADHD symptoms, as measured by the parent-completed version of the SNAP-IV questionnaire, adjusted for pretreatment SNAP-IV score. Secondary outcome measures are: a validated index of behaviour during child’s solo play; teacher-reported SNAP-IV (ADHD scale); teacher and parent SNAP-IV (ODD) Scale; Eyberg Child Behaviour Inventory - Oppositional Defiant Disorder scale; Revised Client Service Receipt Inventory - Health Economics Costs measure and EuroQol (EQ5D) health-related quality-of-life measure. Follow-up measures will be collected 6 months after treatment for participants allocated to adapted-NFPP and IY.

**Discussion:**

This trial will provide evidence as to whether the adapted-NFPP is more effective and cost-effective than the recommended treatment and TAU. It will also provide information about mediating factors (improved parenting and neuropsychological function) and moderating factors (parent and child genetic factors) in any increased benefit.

**Trial registration:**

Current Controlled Trials, ISRCTN39288126.

## Background

Estimates of the prevalence of attention-deficit/hyperactivity disorder (ADHD) vary widely within and between countries. The UK National Institute for Health and Care Excellence (NICE) have estimated that around 5% of school-aged children and adolescents meet the DSM-IV diagnostic criteria for ADHD, equivalent to 366,000 children and adolescents in England and Wales [1]. Attention-deficit/hyperactivity disorder is a debilitating mental health disorder, which is marked by symptoms of inattention, overactivity and impulsiveness that have an early onset, and are age inappropriate, persistent and pervasive [2]. It affects children over their school years and into adulthood [3] and is associated with a number of impairments that impinge on a range of social and health care systems (education, criminal justice, mental health, social services, and so on) [4]. Those with ADHD are at increased risk of delinquency, criminality, educational failure and mental illness [5,6]. Treatment for ADHD typically begins during the middle school years [2] and multimodal approaches are recommended [7]. Stimulant and nonstimulant [7,8] medications are considered the most efficacious treatments and are recommended. However, these have a number of limitations [9-12], including reported side effects [11,13,14] and can give rise to parental concerns about the use of medication for behavioural control [15]. Furthermore, medication on its own might not improve other outcomes, such as social and academic functioning [16]. Nonpharmacological treatments, such as classroom-based behaviour modification or parent-training programmes can also be valuable but appear not to provide the same control of ADHD symptoms offered by pharmacological treatments [17].

To optimize the effects of nonpharmacological therapies, early intervention approaches implemented during the preschool period have been proposed [18]. Such proposals are consistent with the fact that, even though most ADHD cases are first formally diagnosed and treated in middle childhood, ADHD often has its roots in the preschool period [19-21]. In fact, while significant levels of preschool-type ADHD symptoms may not always signal the early ‘onset’ of impairment, numbers of referrals of ADHD before the age of 5 years are growing [1,5,22].

A number of parent-training programmes have been developed to target preschool ADHD [23-25]. The New Forest Parenting Programme (NFPP) is a home-based approach designed specifically to target the core symptoms of ADHD in preschool children. It is delivered on a one-to-one basis [26]. It has been shown in randomized controlled trials (RCTs) to substantially reduce levels of ADHD symptoms and related problems, such as oppositional defiant disorder (ODD) [27,28]. A second approach, the Incredible Years (IY) programme, was originally designed to target children’s oppositional and noncompliant behaviour. It consists of a suite of programmes for parents, children and teachers. While originally, and still mainly, delivered to groups of parents, children and teachers, there is now also a home-coaching model for the parent programme. These programmes, both individual and in various combinations, have been demonstrated to reduce children’s noncompliance and troublesome behaviour and increase positive behaviour [29]. It has also produced generally positive effects on these outcomes with preschool children, including those with or at risk of ADHD [29-32]. A recent adaptation specifically for children with ADHD has shown benefits in terms of parents’ ratings of ADHD symptom reduction [33-37]. The IY programme has been recommended for use with preschool ADHD children in influential guidelines, such as those published by NICE [38], partly on the basis that its group-based nature is thought to be a cost-effective therapy [39].

Despite positive findings from RCTs, there are many barriers to effective implementation of these treatments in everyday practice [40-42], especially with regard to individuals and families traditionally seen as hard to engage and treat. Such factors as the presence of parental mental health problems, the existence of additional child comorbid conditions, cognitive and language problems and the presence of adverse environmental factors need to be addressed if preschool parenting packages are likely to work with those most in need of help. Knowing how to access and engage ‘at-risk’ and ‘hard-to-reach’ children and their families, who may be living in difficult circumstances, is therefore central to the development of an effective early detection and intervention strategy. Previous research indicates that when access is made available, the outcomes for ‘hard-to-reach’ families attending the IY parent programme are as good as those less disadvantaged [43].

The Comparison of Preschool Parenting Interventions (COPPI) trial is the fifth and final project in the 5-year Programme for Early Detection and Intervention for ADHD (PEDIA). This programme of research, funded by the National Institute for Health Research, is being carried out at the University of Southampton in collaboration with Solent NHS Trust, and at two other centres: the University of Nottingham in collaboration with Nottingham City Care and Nottingham County Health Partnerships and the North Staffordshire Combined Healthcare NHS Trust.

Using an evidence-driven approach, the NFPP has been recently adapted to address the specific needs of ‘hard-to-reach’ and ‘difficult-to-treat’ preschool children with ADHD and their families (F McEwan et al., unpublished work) [44]. This has involved extending it from an 8- to a 12-week programme with the inclusion of specific modules to address such problems as parental mental health problems and child comorbid developmental conditions. In this trial, we will evaluate the effectiveness and cost-effectiveness of the adapted-NFPP against IY and treatment as usual (TAU) in a multicentre RCT.

### Aims and objectives

The overall aim of this study is to conduct a large-scale multicentre RCT of the efficacy of the adapted-NFPP by comparing it with (i) a TAU group and (ii) a generic parenting package recommended by NICE (IY) in a sample enriched for potentially hard-to-reach and treat children.

The key objectives are to test whether, for individuals with preschool-type ADHD problems, the adapted-NFPP produces statistically greater beneficial effects in terms of core symptoms of ADHD and other secondary outcomes (general child behaviour problems and parental mental health); to determine whether outcomes are moderated by factors that might be predictive of long-term burden and other potentially important moderators, including genetic factors; to determine whether effects are mediated by (a) improved parenting and (b) improved neuropsychological function; and to determine whether the adapted-NFPP is more cost-effective.

The main research questions are:

1. Does the adapted-NFPP produce statistically greater reductions in ADHD symptoms compared with a TAU group and standard IY 12-week parenting programme in a sample of individuals with preschool-type ADHD problems enriched for ‘hard-to-reach’ and potentially treatment-resistant preschool children?

2. Do these effects extend to other problems, such as child conduct problems?

3. Do they generalize from home to educational settings?

4. Are these effects moderated by (i) factors that might be predictive of long-term burden as well as other putative barriers to treatment efficacy and (ii) genetic factors?

5. Are these effects mediated by (a) improved parenting and (b) improved neuropsychological function?

6. Do benefits persist in the longer term (for example, 6 months after treatment)?

7. Is the adapted-NFPP cost-effective relative to IY in this enriched sample over a 6-month period and does the added value of adapted-NFPP outweigh any additional costs of treatment delivery?

## Methods/design

### Trial design

A multicentre three-arm RCT will be carried out to evaluate the efficacy and cost-effectiveness of adapted-NFPP compared with IY and TAU for preschool children with a research diagnosis of ADHD in a sample enriched to ensure a high percentage of potentially high-burden and difficult-to-treat cases. Participants will be randomly allocated to adapted-NFPP, IY and TAU in the ratio 3:3:1. Randomization will be stratified by participating centre and tranche.

### Study setting

Participants will be enlisted at the three centres participating in the trial: the University of Southampton and Solent NHS Trust; the North Staffordshire Combined Health Care NHS Trust; and the University of Nottingham and Nottingham City Care and Nottinghamshire County Health Partnerships. All centres will run the trial in accordance with good clinical practice guidelines and will work in collaboration with National Institute for Health Research (NIHR) local research networks and with the University of Southampton Clinical Trials Unit (UoSCTU).

### Eligibility criteria

#### Inclusion criteria

Inclusion criteria are: (1) Parents or main caregivers aged 18 years or older (with or without moderate mental health issues); (2) Child aged between 2 years 9 months and 4 years 6 months with significant ADHD-type behaviour (with or without comorbid conditions, such as language and communication difficulties, learning difficulties or behaviour problems); (3) positive screening for ADHD symptoms (score ≥20) based on the parent-reported Werry-Weiss-Peters Activity Rating Scale [45] followed by a score above clinical thresholds on the parent-reported Diagnostic Interview Schedule for Children Version 4 (DISC-IV) ADHD Scale [46]; that is, six or more symptoms on either the inattention subscale or the hyperactive/impulsivity subscale, or combined, but in either case with a rating of impact or impairment.

#### Exclusion criteria

Child exclusion criteria are: (i) diagnosis of autism; (ii) being in foster care without a long-term plan in that foster placement; (iii) extreme learning difficulties, as defined by a score below an age-appropriate developmental level for more than 6 out of 12 items taken from four scales of the Parent Involvement Project (PIP) Developmental Scales [47] including: physical development, three items; development, four items; eye-hand co-ordination development, one item; development of play, four items; (iv) very poor or no language as defined by a score below an age-appropriate developmental level for more than three out of six items taken from the Language Development Scale of the PIP Developmental Scales [47]. Parent exclusion criteria are: (v) no working knowledge of English; (vi) serious mental illness (for example, psychosis, extreme learning difficulties, manic depressive disorder); (vii) having a child on the Child Protection Register.

### Interventions

#### Arm A: Adapted-NFPP

The original 8-week individually delivered home-based NFPP was developed as a specialized psychological intervention for preschool children and focuses on reducing the core symptoms of ADHD [48]. The value of early intervention is demonstrated in three NFPP trials [26-28], which have shown it to be effective in reducing ADHD symptoms and conduct problems. Short-term effects on parental mental health and the quality of mother-child interactions were also observed [26,27]. This programme includes components of psychoeducation of parents about ADHD and teaches strategies of proactive parenting in the context of a child with ADHD. It aims to enhance the mother-child relationship through play. It also includes an attention and organizational training component to target the underlying neuropsychological basis of the condition (for example, poor concentration and working memory and inability to cope with delay) using games played together by the preschool ADHD child and the child’s mother. The original trial of this programme in a RCT had an effect size of 0.87 for ADHD symptoms [27], while a recent smaller RCT had an effect size of 1.96 [26]. The adapted-NFPP is based on the original NFPP but modified to be potentially more effective with treatment-resistant and hard-to-reach families and children on the basis of qualitative research [49] and piloting carried out prior to the start of the current trial (F McEwan et al., unpublished work). The 12-week programme provides the time to deliver the original 8-week programme at a slower pace. Additional modules are included to address (a) the child’s sleep problems, learning difficulties and speech and language problems and (b) parental mental health problems, learning difficulties and ADHD. A component on mindfulness for parents has also been added, as has training in motivational techniques and the use of social stories. The adapted-NFPP will be carried out in weekly sessions of approximately 1.5 hours’ duration over a 12-week period. Course materials provided during the course of the programme include handouts, a DVD (adapted for preschool children), an audio CD and other resources (see Appendix 1).

#### Arm B: Incredible Years

The IY programme is a series of programmes for parents, children and teachers and includes a suite of parenting programmes focused on strengthening parenting competencies (monitoring, positive discipline, confidence) and fostering the parent–child relationship, as well as targeting involvement in children’s school experiences, in order to promote academic, social and emotional competencies and reduce conduct problems. The IY parenting programmes [50], as recommended by NICE [38], have been developed to help parents prevent or manage oppositional and challenging behaviour through the use of reinforcement and have been shown to be effective in improving parenting skills, reducing conduct problems and improving parent–child relationships [29,35,51]. A modified version of the IY being used in the current trial has also shown some value in the treatment of preschool children displaying early signs of ADHD [33,36,37]. The parenting programmes are recommended by the American Psychological Association Task Force as meeting accepted criteria for empirically supported mental health intervention for children with conduct problems. They are also one of the few programmes identified in the University of Colorado Blueprint classification of programmes with good evidence for violence prevention [52]. The techniques used to help parents acquire new skills are facilitator-led group discussion, brainstorming, videotape modelling, role play and rehearsal of the taught intervention techniques within groups and through homework. A number of skills are taught in weekly sessions, usually over a period of 12 weeks or more. Parents are shown how to use: (i) play and child-centred activities to promote a positive relationship with their child; (ii) praise, reward and incentive to promote appropriate child behaviours; (iii) effective limit setting and clear instruction; and (iv) the use of strategies to manage noncompliant behaviour. Course materials include handouts, CDs, books and prizes, which are awarded during the course of the programme. Parents who miss a session receive weekly calls and also, where possible, visits from a therapist to encourage the parent and to monitor progress. This is an 8-part programme, with each part building on the previous one, and is designed to support parents by strengthening positive and nurturing parenting skills (see Appendix 2). It is carried out in weekly sessions of approximately 2 to 2.5 hours’ duration over a 12-week period.

#### Arm C: TAU

For those parents randomized to the TAU group, measures will be collected before and after the 12-week programme. Measures will not be collected from the TAU group 6 months after treatment, as with the adapted-NFPP and IY participants. After final data collection, parents in the TAU group will be offered individual home-based treatment with adapted-NFPP or a place in an IY group-based programme.

### Programme fidelity

Strategies for ensuring fidelity are incorporated as integral parts of the two parenting programmes, including training and monitoring procedures. In addition, a common approach will also be adopted to assess fidelity and integrity as required by each programme, so that these factors can be compared across treatments and their effects on outcome assessed. Procedures for optimizing fidelity relate to the selection, training and supervision of therapists and the skills to be acquired by therapists. The therapists for both programmes come mostly from a health background and are nurses with a background in child and adult mental health services (CAMHS), paediatrics or health visiting; psychologists with a background in CAMHS or behavioural management; family support workers with a background in CAMHS; or senior social workers with experience in children services. As initial training, therapists delivering both programmes will attend a residential training course focused on the techniques necessary to deliver the programme. The basic 3-day IY parent group leader workshop teaches leaders how to use the programme as preventive early intervention in a range of settings. Attending this workshop is the first step in becoming a certified group leader. Training covers three parent programmes, for parents of toddlers aged 1 to 3 years, parents of preschool children aged 3 to 6 years and parents of school-aged children 6 to 8 years old. Both sets of therapists will have ongoing supervision both locally and centrally throughout the time of the trial using face-to-face supervision, by telephone and monitoring of videotaped sessions recording their work with families. Training and supervision of IY therapists and leaders is overseen by practitioners with extensive experience of delivering IY and a clinician (JH) with extensive experience in working with the IY set of programmes and their development. Training and supervision of adapted-NFPP therapists is overseen by a clinician (MT) and a consultant nurse specializing in ADHD (CLB), both with extensive experience in conducting the NFPP and its development.

### Fidelity measures

Session checklists and evaluation forms will be completed by therapists. Three instruments will also be used to monitor process and treatment fidelity across both programmes and their moderating effects on outcomes. Process fidelity will be measured using a modified version of the Leader Observation Tool (LOT) after treatment is finished [53]. The Working Alliance Inventory [54] will be used to look at aspects of the parent-therapist relationship during therapy. The impact of treatment on the family will be measured using the Family Strain Index [55].

### Study measures

#### Screen and diagnostic interview

Three measures will be administered to determine the eligibility of parents and their children for inclusion in the study: the Werry-Weiss-Peters Activity Rating Scale (WWP) [45]; the DISC-IV ADHD Scale [46] and the PIP Developmental Scale [47]. Scores on the WWP will determine whether the child has ADHD symptoms at a significant level of severity to proceed with the administration of the DISC-IV-Parent preschool ADHD diagnostic interview. Items on the PIP Developmental Scale will indicate the presence of poor or no language and severe child learning disability or poor development.

#### Primary and secondary measures

The primary outcome measure is the SNAP-IV-P ADHD Scale (parent-completed), which will be collected at the three time points and records the frequency of occurrence of ADHD symptoms [56]. Secondary outcome measures include a child observation measure carried out by the research fellow using the Child’s Solo Play Index [27]; the teacher-completed SNAP-IV-T ADHD Scale; the teacher and parent-completed SNAP-IV ODD scale [56] and the Eyberg Child Behaviour Inventory [57], both of which are measures of noncompliant and oppositional behaviour

#### Economic costing

Economic measures include the Revised Client Service Receipt Inventory (R-CSRI) [58] and the parent-reported EQ(5D) and proxy-parent-reported child measure [59].

#### Mediators

Factors that might play a mediating role in the effectiveness of the parenting programmes will be investigated and will be collected at all time points. Measures employed will include the expressed emotion five-minute speech sample (FMSS) [60]; the Global Impressions of Parent–child Interactions (GIPCI) observation measures, which include ‘Jigsaw’, ‘Tidy-up’ and ‘Free play’ [61,62]; the ‘Cookie delay’ task, which is a measure of inhibition [63], and the parent-completed General Health Questionnaire [64].

#### Moderators

Factors that might play a moderating role include the Current Symptoms Scale [65], which is a measure of parental ADHD symptoms. Saliva samples will also be collected to investigate parent and child gene polymorphisms related to the ADHD phenotype including *DRD4*, *DRD2*, *5HTT-LPR*, *COMT*, *MAO-A* and *OR-A* and their impact on the effectiveness of the parenting programme.

The possible role of the PARI (Preschool ADHD Risk Index) in moderating the effectiveness of the parenting programmes will also be examined. As part of the overall 5-year PEDIA programme of research, the PARI will be developed based on the outcomes of a longitudinal study of adult outcomes of preschool hyperactivity. A range of child behavioural and developmental factors will be considered, together with a range of maternal and demographic factors. A demographic questionnaire will be administered at baseline for the purposes of PARI analysis and to characterize the sample. The parent-completed Current Symptom Scale, a parental ADHD symptom measure, and the Emotionality, Activity, Sociability and Impulsivity (EASI) questionnaire, a measure of child temperament, will also be administered at baseline only and used for the purposes of the PARI analysis, if these factors are found to contribute to high risk and burden in the ongoing longitudinal study.

A more detailed description of all study instruments is included in the section on data collection methods.

### Participant timelines

Outcome measures are collected at pretreatment (T1), post-treatment (T2) and, in the case of those participants allocated to Arm A or Arm B, also at 6 months post-treatment (T3). A time schedule of enrolment, interventions and measures is presented in Table 1.

**Table 1:** Enrolment, interventions and assessments

**Contact and home visits**	**Screening or diagnostic interview**	**Consent and TENALEA allocation**	**Pretreatment (T1) visit**	**Induction visit by therapist**	**Intervention: a-NFPP (home) IY (local hall)**	**Post-treatment (T2) visit (TAU = end of study)**	**6 month follow-up (T3) visit (a-NFPP/IY = end of study)**
**Timeline**	Week 1 to Week 10			Week 11 to 12	Week 13 to 24	Week 25 to 28	Week 50
**Enrolment**							
Participant informed consent	✓	✓					
**Eligibility**							
WWP (child hyperactivity); parent-completed scale	✓						
PIP developmental scales; research-fellow-administered scale	✓						
DISC-IV ADHD scale, child; research-fellow-administered interview	✓						
**TENALEA allocation**			✓				
**Interventions**							
Arm A: a-NFPP	✓	✓	✓	✓	✓	✓	✓
Arm B: IY	✓	✓	✓	✓	✓	✓	✓
Arm C: TAU	✓	✓	✓			✓	
**Measures**							
**Primary outcome**							
SNAP IV-P ADHD scale; parent-completed questionnaire			✓			✓	✓
**Secondary outcomes**			✓			✓	✓
Child’s solo play; observation measure, research-fellow-completed			✓			✓	✓
SNAP IV-T ADHD scale; teacher-completed questionnaire			✓			✓	✓
SNAP IV-P and T ODD scale: parent- and teacher-completed questionnaires			✓			✓	✓
ECBI (ODD measure); parent-completed questionnaire			✓			✓	✓
CSRI-Revised (economic costs); research-fellow-administered questionnaire			✓			✓	✓
EQ-5D QOL: Parent and parent proxy; parent-completed questionnaires			✓			✓	✓
**Mediators**							
FMSS (maternal expressed emotion); research-fellow-rated			✓			✓	✓
GIPCI, child/parent observation measure; research-fellow-rated			✓			✓	✓
Cookie delay task, child observation measure; research-fellow-rated			✓			✓	✓
GHQ - maternal; parent-completed questionnaire			✓			✓	✓
**Moderators**							
CSS (parental ADHD); parent-completed questionnaire			✓				
Preschool ADHD Risk Index; research-fellow-scored index			✓				
EASI (child temperament); parent-completed questionnaire			✓				
Demographic questionnaire; parent-completed questionnaire			✓				
Genetic testing (saliva sample); self-collected, parent & child						✓	✓
LOT; research-fellow-rated							✓
WAI-S; parent- and therapist-completed questionnaires			✓			✓	
FSI; parent- and therapist-rated			✓			✓	

a-NFPP: adapted New Forest Parenting Programme; CSRI-R: Revised Client Service Receipt Inventory; CSS, Current Symptoms Scale; DISC-IV, Diagnostic Interview Schedule for Children Version 4; EASI, Emotionality, Activity, Sociability and Impulsivity; EE, expressed emotion; ECBI, Eyberg Child Behaviour Inventory; EQ-5D, EuroQol - 5 Dimensions; FMSS, five-minute speech sample; FSI, Family Strain Index; GHQ, General Health Questionnaire; GIPCI, Global Impressions of Parent–child Interactions; IY, Incredible Years; ODD, oppositional defiant disorder; PARI, Preschool ADHD Risk Index; PIP, Parenting Intervention Project; SNAP-IV-P, Swanson, Nolan and Pelham Version 4 parent version; SNAP-IV-T, Swanson, Nolan and Pelham Version 4 teacher version; TAU, treatment as usual; WAI-S, Working Alliance Inventory, Short Form; WWP, Werry-Weiss-Peters Activity Rating Scale.

### Sample size

The trial is powered for two comparisons of interest.

Primary question: adapted-NFPP versus Incredible Years (Arm A vs Arm B).

Is adapted-NFPP superior to IY in terms of reductions in parent-rated ADHD symptoms? Assuming a conservative estimate of effect size difference of 0.4 standard deviation between adapted-NFPP and IY, an intraclass correlation of 0.08 for parents treated in the same IY groups and a drop-out rate of 10% (considerably higher than in the original trials) it is estimated that 141 individuals will be needed per treatment arm to provide sufficient power to test the hypothesis that the adapted-NFPP is superior to IY in terms of reduction in ADHD symptoms (total *N* = 282). This equates to a difference of mean 0.28 and standard deviation 0.7 on the SNAP-IV-P ADHD rating scale (the primary outcome) using a 5% two-tailed test of significance and 80% power. A design effect of 1.28 has been calculated from the formula:

$$1+\left(m-1\right)p$$

in the CONSORT (Consolidated Standards of Reporting Trials) statement for nonpharmacological interventions [66].

Secondary question: adapted-NFPP versus treatment as usual (Arm A vs Arm C).

Is the adapted-NFPP superior to TAU in terms of reductions in parent-rated ADHD symptoms? Again assuming a conservative estimate of effect size difference between adapted-NFPP vs TAU, a group of 141 adapted-NFPP and 47 TAU will give 81% power to detect an effect size of 0.5 on the SNAP-IV-P ADHD score (equating to a difference of mean 0.35 and standard deviation 0.7) with 5% two-tailed significance. No intraclass correlation has been applied for this calculation, as both adapted-NFPP and TAU will be delivered individually.

On this basis, each of the three centres will recruit approximately 110 families with a preschool-aged child (age 2 years 9 months to 4 years 6 months) with high levels of ADHD symptoms (total *n* = 329) into the trial. Families will be randomized with intention to treat to either (i) adapted-NFPP as an individually delivered, home-based parenting programme (*n* =141, leaving 127 for final analysis with 10% drop-out); (ii) IY delivered in groups of eight (*n* = 141, leaving 127 for final analysis with 10% drop-out); or (iii) TAU (*n* = 47, leaving 42 for the final analysis with 10% drop-out).

### Recruitment

The plan for recruitment takes into account that (a) two research fellows will be available at each centre to conduct screening and the diagnostic interview, recruit participants to the study and complete T1 to T3 visits for each tranche and (b) two pairs of therapists will be employed at each centre to cover the two parenting programmes. Given a target recruitment of *n* = 329 participants (110 participants per centre) and factoring in school holiday periods, it is estimated that five or six parenting programme tranches will be needed, with 18 to 22 parents enlisted per tranche across centres. An allocation ratio of 3:3:1 will mean that approximately eight parents will be allocated to each pair of therapists. Two NFPP therapists will each work on a one-to-one basis with four parents over the 12-week period of the programme and two IY therapists or leaders will conduct a group-based programme in a local hall with eight parents, whose partners may also attend the group.

The collaboration of local agencies and services acting as participant identification centres across each of the sites will be sought to help achieve recruitment targets for each tranche of parenting programmes. Practitioners and clinicians across agencies will make study enlistment packs available to parents. These packs contain a letter to the parent, an information sheet, an expression of interest form, a consent to screen form and a freepost envelope for the return of the expression of interest and consent forms to the research office. Posters, radio advertisements and social media will also be used to recruit a broad range of parents to the study. For the purpose of ensuring sufficient numbers of hard-to-reach and potentially difficult-to-treat families, the sample will be enriched by targeting a range of different referral sources and using previously developed outreach strategies to increase engagement of these families. Multiple recruitment routes will be used at each of the centres participating in the trial; these will include health visiting and Sure Start professionals; speech and language clinics; community paediatric clinics; and CAMHS. To determine the proportion of ‘hard-to-reach’ and potentially treatment-resistant families participating in the trial, the sample will be characterized using a number of factors that have been associated with such families. These factors will include: socioeconomic status; education; employment; number and age of siblings residing in the home; ethnicity; single parent status; age of parent at time of birth; parental mental health problems or ADHD; child language and communication problems and the presence of other child comorbid conditions.

## Informed consent

In the 10-week period prior to the start of each parenting programme tranche, two research fellows in each area will have responsibility for obtaining written informed consent to administer screening and diagnostic measures to parents, usually at their homes or some other convenient location or, if necessary, by phone. Standard informed consent procedures will be followed both for the administration of screen and diagnostic interview measures and, if the parent is eligible and willing, for participation in the full study. If the parent is not eligible, the parent will be informed and this will be confirmed in writing to the parent and the parent’s clinician or practitioner where appropriate. If the measures indicate that (1) all inclusion criteria have been met and (2) that in the event of being allocated to IY, the parent would be available to attend an IY programme on a fixed day in a local centre or hall where crèche facilities will be made available, the parent and child will be eligible for inclusion in the full study. A screen and diagnostic interview case report form (CRF) will be completed by the research fellow, indicating that all inclusion criteria were met together with the parent or main caregiver scores for all completed measures.

If the parent is willing to participate, the research fellow will arrange to visit the parent’s home. At the T1 visit, written informed consent to participate in the full study will be obtained and T1 measures will be collected. Protocol assessments will be performed according to the schedule in Table 1. After consent is obtained and the visit has been completed, a T1 CRF will be completed by the research fellow to record the measures completed at the visit and the scores for these measures.

The research administrator will then liaise with the UoSCTU to complete the process of randomization and the participant will be randomized to an arm of the study. General practitioners will be informed of the parent’s participation both at the start and end of the study. All participants will be informed that they can withdraw from the study at any time without their rights being affected. In the case where consent to treatment is withdrawn but the participant agrees to remain in the research study, the participant will be followed to completion.

### Allocation and blinding

After consent has been received, the participant will be randomized into an arm of the study by the UoSCTU using the online TENALEA system [67]. Randomization procedures will be carried out prior to the start of each parenting programme tranche and will take place during normal working hours (Monday to Friday, 9 a.m. to 5 p.m.). Only the trial co-ordinator (DMC) and designated research administrator staff at each centre will be responsible for liaising with the UoSCTU in randomizing participants. When possible and to help maintain blindness, groups of eight or more participants within each centre will be randomized together. Upon receipt of an electronic file from each centre, designed by the UoSCTU to provide participant IDs and initials, centre attended and other details necessary for TENALEA system randomization, the UoSCTU will carry out randomization. Parents will be block randomized to adapted-NFPP, IY or TAU arms in the ratio 3:3:1. Automated email confirmation of randomization will be sent to the authorized individual at each centre. Details of the randomization will then be added to the screen and diagnostic interview CRF by the research administrator. A copy of this CRF, together with the T1 CRF, will be checked and verified by the centre principal investigator and sent by courier or, in the case of the Southampton centre, delivered by hand to the UoSCTU, along with a copy of all measures collected at these two visits. Data measures will be gathered and CRFs completed and processed in a similar manner at T2 and T3 visits.

Participants in each centre will be informed by designated research staff as to which arm of the study they have been allocated and this will be confirmed in writing. In the case of parents randomized to the TAU group, parents will be offered a place on a parenting programme in the community to commence approximately 4 weeks after T2 data collection, which will be ‘end of study’ for TAU participants. Those participants allocated to adapted-NFPP and IY will also be asked to complete T3 measures 6 months after treatment. Research fellows will remain blind to treatment allocation. Research fellows will inform the participant when arranging home visits at T2 and T3 data collection that this blindness should be maintained on their visit. Any breaches of blindness will be recorded. The screen and diagnostic interview CRF containing allocation details will be filed separately from other documents contained in participant files to which the research fellows will have access, as will all other such documents.

### Data collection methods

Data collection will take place at the parent’s home at each of the three time points. All research fellows will have a degree in psychology and will be trained in the administration of all measures. The majority of measures are self-completed (on paper) by the parent, as indicated in Table 1. A description of study instruments are outlined below together with details of reliability and validity, where available.

#### Screen and diagnostic interview

##### Werry-Weiss-Peters activity rating scale (WWP: [45])

The WWP is a 27-item parent-completed questionnaire measuring hyperactivity. This has been shown to identify the top 15 to 18% of the population using a score of 20 as a cut-off [68]. Psychometric properties have been reviewed by Barkley [69], who reported discrimination between hyperactive and normally developing children to be good. The interparent agreement has also been found to be good (*r* = 0.82) [70]. The WWP is easy to complete and has been shown to have high levels of internal consistency, to correlate with other measures of hyperactivity and to identify children who have activity problems 5 years later [71].

##### Diagnostic interview schedule for children – version 4 (DISC-IV - ADHD Scale [46])

If a score of 20 or more is reported on the WWP, the DISC-IV will be administered to the parent or main caregiver. The DISC-IV uses criteria contained in the DSM-IV and enquires about hyperactive and inattentive symptoms and impairment in both home and school settings. The standard DISC-IV impairment algorithm requires moderate impairment in at least one area of functioning related to ADHD symptoms, as judged by parental responses. Impairment on the DISC is defined by the degree to which the symptoms have (a) caused distress to the child; (b) affected relations with caregivers, family, friends or teachers; or (c) affected school functioning. The DISC has been shown to have moderate to substantial test-retest reliability and internal consistencies. Training in administration and scoring of this measure was overseen by the clinical lead (MT), the trial co-ordinator and other researchers with extensive experience in its administration.

##### Parent Involvement Project (PIP) developmental scales [47]

If DISC-IV inclusion criteria are met, the PIP developmental scales will be administered by the research fellow. This developmental checklist is widely used by educational psychologists as either an interview schedule with the parents or an observation schedule. It gives a profile of the child in five different areas: physical development, social development, eye-hand co-ordination development, development of play and language development. The chart also suggests the age that children will normally develop these skills and has age norms up to age 4 years. In this study, it will be used as an interview schedule with the mother and it will give an indication of any possible developmental delay. An educational psychologist was consulted regarding the scoring of these scales and training in the administration of this measure by the research fellow was carried out by the clinical lead (MT).

#### Outcome measures

##### SNAP-IV parent (SNAP-IV-P: 56]) and teacher scales (SNAP-IV-T: 56])

The self-completed SNAP-IV-Parent ADHD Scale is the primary outcome measure in this trial with the teacher-completed SNAP-IV-T ADHD Scale and the SNAP-IV-P and T ODD Scale used as secondary outcome measures. The Multimodal Treatment Study of Children with ADHD (MTA) version of the SNAP-IV is used in this trial [72]. The 26 items include 18 ADHD symptoms (nine inattentive, nine hyperactive or impulsive) and 8 ODD symptoms, as specified in the DSM-IV. Items are rated for frequency by the parent or teacher on a 4-point scale (0 = not at all to 3 = very much) and average ratings per item are then calculated for each subscale. Bussing et al. [56] examined the psychometric properties of the SNAP-IV-P and found that the internal consistency for overall parent ratings was 0.94 (inattentive 0.90; hyperactive or impulsive 0.79, ODD 0.89). Internal consistency for the SNAP-IV-T was 0.97 (inattentive 0.96, hyperactive or impulsive 0.92, ODD 0.96). Interrater reliability between parent and teacher ratings was 0.49 for inattention, 0.43 for hyperactivity or impulsivity and 0.47 for ODD and all were statistically significant (*P* < 0.001).

##### Eyberg child behaviour inventory (ECBI: [57])

The ECBI is a parent-completed 36-item inventory for the assessment of problem behaviours in children aged 2 to 16 years. It includes a seven-point intensity scale (never to always) that measures the frequency of each problem behaviour and a yes-no problem scale that identifies whether the behaviour is currently seen as a problem. The ECBI can be used both as a clinical screening measure for identifying and treating externalizing problems in children and as a measure of treatment outcome. Cut-offs representing high-risk children are 127 for the problem scale and 11 for the intensity scale. The ECBI has been shown to correlate well with independent observations of children’s behaviours and can differentiate between clinic-referred and nonclinical populations. Reliability coefficients for the ECBI scales range from 0.86 (test-retest) to 0.98 (internal consistency) [57,73].

##### Child’s solo play [27]

This independent observation measure, which is carried out by the research fellow during 5 minutes of solo child play with a standard activity toy, is an important secondary outcome measure. The ‘Little People Animal Sounds Zoo’ is a multipurpose toy that includes a number of different activity zones (for example, water slides, a ticket office and a tree cave). Patterns of attending to and switching from one activity to another during independent play are measured and an index of attention or engagement is calculated (time on task divided by total number of switches from zone to zone). High index scores represent more attention and less switching. This measure has high test-retest reliability (Pearson *r* = 0.81) and interrater reliability (Pearson *r* = 0.76) and good validity, differentiating, as it does, children with ADHD from those without. Research fellows are trained in the administration and scoring of solo play by one of the principal investigators (DD), who has extensive experience in the development and scoring of this measure.

##### Revised client service receipt inventory (CSRI-R: 58])

The CSRI-R allows the collection and combining of health economic data in a retrospective format from a range of different sources using a range of different approaches (interview, health records, and so on). In this study, a 3-month recall window or ‘since the last CSRI’ will be used for the interview section. The interview section conducted by the research fellow asks about the parental background and whether the child’s behaviour has affected career prospects and has had an impact on the finances of the family. There is also a section on use of services, including any major service use at any point in the young person’s life. The CSRI has good face validity [58] and test-retest reliability and has been used in several evaluations of care for children with needs related to mental health. Instruction in the completion of this measure will be given by experienced health economists.

##### EQ-5D [59]

The EuroQol or EQ-5D is a standardized questionnaire for use as a measure of health outcome. The EQ-5D-3 L (five dimensions, three levels) used in this study consists of the descriptive system and the EQ-5D visual analogue scale (EQ-5D VAS). The descriptive system comprises five dimensions: mobility, self-care, usual activities, pain or discomfort and anxiety or depression. Each dimension has three levels, no problems, some problems and extreme problems, and the respondent is asked to tick the box against the most appropriate statement in each of the five dimensions. The VAS records the respondent’s self-rated health on a vertical, visual analogue scale (0 to 100) with endpoints representing the worst and best imaginable health states. Both the self-completed parent- and proxy-parent-reported child measures are used in this trial. This is a generic measure, applicable to a wide range of health conditions and can be used in the clinical and economic evaluation of health care. The parent-completed measure is widely used and has shown moderate levels of validity. The parent-proxy measure has not been sufficiently validated, particularly with younger children. However, Matza and colleagues [74] have looked at the parent-proxy ratings of children with ADHD in the USA and UK and reported that it was able to detect impairment in these children. The index and the VAS also showed significant correlations with ADHD symptoms using DSM-IV criteria for ADHD. Matza et al. concluded that parent-proxy ratings are feasible and valid for use as part of an overall health outcomes assessment in clinical studies of childhood ADHD [74].

#### Mediators

The following measures allow investigation of the possible role of positive and constructive parenting and scaffolding, improved maternal mental health and improved inhibition with greater delay tolerance by the child in mediating the impact and effectiveness of the parenting intervention.

##### Expressed emotion (EE): five-minute preschool speech sample (FMSS: [60])

The parent is asked to speak for exactly 5 minutes about his or her child, his or her feelings and thoughts about the child, and how they get along together. The parent is to use his or her own words without any interruptions from the interviewer. The parent’s response is taped. Parental EE is rated on the initial statement, warmth, emotional overinvolvement, relationship, positive and critical comments. A coding manual is used for the FMSS. An overall score can be given for the level of EE with a high level indicating a negative influence. The measure has shown acceptable code-recode and interrater reliability and adequate test-retest reliability. It also demonstrated to have acceptable validity, being associated with less affection and greater maternal direction during play interaction, and discriminating between the parents of preschool children with symptoms of ADHD and those without such symptoms [60]. Training of research fellows in the administration and scoring of the FMSS will be overseen by the author of this measure (DD).

##### Global impressions of parent–child interactions - revised (GIPCI-R: [61,62])

This measure involves the direct observation of mother-child interactions while completing three tasks, each of 5 minutes’ duration: ‘jigsaw’, ‘free play’ and ‘tidy-up’. The 15 minutes of parent–child interaction are videotaped and coded at a later time using the GIPCI coding manual. Child items rated are: respect, destruction, disruptive, noncompliance, social skills, valence and disconnection. Parent items rated are valence, responsiveness, warmth, praise, enjoyment, scaffolding, effectiveness, aggression and criticism or punishment. The measure generates summary global ratings (1 to 5) for both parent and child behaviours with a higher score indicating a more positive outcome. Thompson et al. [26] found adequate interrater reliability for child scores across all item codes (0.62; range 0.48 to 0.77) and for parent scores (0.64; range 0.48 to 0.79). There was also good internal consistency for child and parent scales (0.84; 0.87). Test-retest reliability was adequate for the parent measure (*r* = 0.50) but low for the child measure (*r* = 0.20). Training in administration and scoring will be carried out by the clinical lead in the trial (MT).

##### Delay of gratification: cookie delay task [63]

This task involves placing an edible treat under one of three upturned transparent cups and asking the child to wait for a signal before retrieving the treat. Eight trials are given in a pseudorandom order with delays of between 5 and 30 seconds. The scoring is 0 = not inhibited, 1 = partially inhibited and 2 = fully inhibited, giving a possible range of scores 0 to 16. The task has been shown to be reliable and to discriminate between hard-to-manage preschoolers and their peers [75].

##### General health questionnaire (GHQ: [64])

The parent-completed GHQ was developed as a screening instrument for use in primary care settings to identify those likely to have or be at risk of developing psychiatric disorders. It is a measure of common mental health problems, such as depression, anxiety, somatic symptoms and social withdrawal. This trial employs the 12-item version of the GHQ, which has been shown to be consistent and reliable across time. Items are scored from 0 to 3, with a total score ranging from 0 to 36, where a lower score indicates health and a higher score indicates illness. This measure is widely used and has shown adequate reliability and validity over a number of studies.

#### Moderators

The possible moderating role of high-risk factors, including the presence of maternal ADHD symptoms, and other indicators of poor outcome and long-term high burden will be investigated. Measures will also be used to monitor process and treatment fidelity across both parenting programmes.

##### Current symptoms scale (CSS: [67]

The CSS is an 18-item questionnaire designed to assess ADHD symptoms in adults and is based on the DSM-IV definition of symptoms of ADHD. The CSS self-report scale requires adults to rate the frequency of their own symptoms over the previous 6 months on a 4-point scale ranging from 0 (rarely) to 3 (very often). A score of nine symptoms or more experienced as ‘often’ or ‘very often’ is used as the standard cut-off to identify adults at risk of clinical problems of ADHD. The scale has been shown to be correlated with spousal, parental and cohabiting partner ratings of symptoms. It comes with a manual for hand scoring, has age- and sex-specific norms and has good psychometric properties, including acceptable levels of internal consistency and excellent test-retest reliability.

##### Preschool ADHD risk index (PARI)

The aim of one of the projects in the 5-year Programme for Detection and Early Intervention for ADHD is to develop the PARI, a way to identify preschool children with ADHD most at risk of poor outcome and high burden. This is based on a prospective follow-up of a large sample of individuals who were identified as having symptoms of ADHD at a preschool age in the 1990s but are now entering late adolescence or early adult life. The risk index is derived from a statistical process involving (i) identification of those children with high levels of ADHD symptoms at 3 years who fall into high- and low-burden groups and (ii) the use of logistic regression to model child, parental and socioeconomic predictors of poor outcome and high burden in the sample of children. Ten parameters will be included in the model: initial ADHD symptom levels; socio-emotional problems; temperament; language delay; developmental delay; maternal mental health; maternal age; parental marital status; perinatal risk and social class. This information will be obtained from a number of sources, including birth and patient records, a demographic questionnaire and the current trial measures (for example, maternal mental health). The temperament measures will be taken using the EASI scale, a 10-item scale measuring emotionality (five items) and shyness (five items), giving a score out of 5 for each subscale [76]. The index so developed and used in the current study will include the significant predictors of high burden weighted in relation to the strength of their contribution to the regression model. The final form taken by the index which will be used will be based on data collected from the ongoing PARI study and is not currently known at present.

##### Leader Observation Tool (LOT: [53])

Process fidelity will be measured using a modified version of the LOT after treatment is finished. This revised version is adapted from the LOT tool used to successfully monitor the fidelity of the IY, which is delivered to parents in a group format. Random sessions from the videotaped IY and adapted-NFPP therapy sessions will be observed and coded by an independent observer, who will score adherence to content week by week as well as the process by which the programme is delivered. The LOT has high internal reliability and good code-recode and interrater reliability. Evidence of concurrent validity has also been obtained.

##### Working alliance inventory, short form: parent and therapist versions (WAI-S: [54])

The WAI-S is a 12-item self-report measure of working alliance. The working alliance is an important part of the process of therapy, and looks at the alliance of task, goals and relationship of therapist with parent and vice versa. Most studies suggest that this alliance is usually forged within 2 to 4 weeks of the beginning of therapy. Two versions of the WAI-S are available: a client version and a therapist version. In this trial, both versions will be completed by the parent and therapist in weeks 6 and 12 of the parenting programme with each form being treated as confidential and delivered to the research office in a sealed envelope by the therapist. The authors of this measure found a correlation with outcome. The WAI-S has three subscales: goals, tasks and bond. Each subscale is scored on a 7-point Likert-type scale ranging from 1 (never) to 7 (always) and has four nonoverlapping items. Subscale scores can range from 4 to 28, to produce a total score of 12 to 84 with higher scores reflecting more positive ratings of working alliance. Internal consistency estimates of the three subscale scores, based on an initial validation sample of 124 pairs of parents and their therapists, ranged from 0.90 to 0.92 (client version) and 0.83 to 0.91. Internal consistency estimates of the total scores were 0.98 (parent version) and 0.95 (therapist version [54].

##### Family Strain Index (FSI: [55])

Impact of treatment on the family will be measured using the FSI questionnaire at the start and end of the parenting programme. This six-item questionnaire is designed to measure two primary aspects of stress and demand on parents and families, specifically, the ‘emotional’, and ‘restrictiveness’ experiences of living with a child with ADHD. Parents will be asked to rate the frequency of occurrence of each of six items over the past 4 weeks on a 5-point scale scored 0 (never) to 4 (always) with a total score in the range 0 to 24. This measure will be used at baseline and at the end of therapy.

The measure has been shown to have excellent internal consistency across the six items (Cronbach’s α = 0.83 to 0.87) and for the full scale (Cronbach’s α = 0.87).

### Statistical analysis

The main analyses will be performed using the intention-to-treat population. All results will be reported according to the CONSORT statement for nonpharmacological interventions [66]. The primary endpoint for the comparison between adapted-NFPP and IY will be the average ADHD score on items 1 to 18 of the SNAP-IV-P at post-treatment. The primary analysis of this endpoint will adjust for baseline SNAP-IV-P scores using a mixed-effect regression model [77] with treatment arm and time (pre- and post-treatment) as the fixed effects and therapist as a random effect. Subject to approval, this analysis will also include trial centre and recruitment tranche as randomization stratification factors. These models are especially valuable when used with longitudinal and trial data derived from parallel arms. A similar secondary analysis comparing adapted-NFPP and IY will be carried out 6 months after treatment. Secondary outcome measures, including the Child’s Solo Play Index, ECBI (Parent), ADHD SNAP-IV-T and SNAP-IV-T, together with SNAP-IV-P ODD scores, will be analyzed in the same way as SNAP-IV-P ADHD.

Subject to approval, a per-protocol analysis will also be performed. The parent will be considered to have completed the parenting programme if he or she had completed at least 8 of the 12 programme sessions. In the case of the IY programme, this will be either by attending the group or by receiving individual home-based sessions for any missed sessions.

#### Therapist effects

Four or more therapists (depending on numbers recruited) will work in each of the three study centres with two therapists working on each intervention. The mixed effects regression models will include therapist as random effects within these models, allowing for the extent to which treatment effects differ between therapists and also accounting for the way in which participants are nested by therapist. Even when the difference between treatment effects can be assumed to be identical for all therapists, the mixed model can improve the precision of the treatment estimates by taking appropriate account of the different therapists in the analysis.

#### Missing data

Drop-out in this trial is defined as noncompletion of measures at the end of the 12-week parenting programmes. A drop-out rate of 10% has been estimated and the rates of drop-out across the three arms will be compared using analysis of variance. The randomness of missing data resulting from drop-out will be explored across treatment arms and, if random, standard mixed-effect regression model procedures will be employed. If data loss is not random, the modelling strategy will be based on Yang and Shoptaw [78], which involves imputation of nonignorable missing data due to drop-out using different models. The effect of using several different models will be compared to estimate imputed values. This method allows an exploration of the range of possible effects of missingness and provides the most conservative estimate of treatment effects, taking into account missing data due to drop-outs.

#### Mediational analysis

Exploratory mediational analysis will examine the extent to which changes in ADHD symptoms are determined by the effects of the treatment package on parents (more positive and constructive parenting or improved maternal mental health) and on children (improved inhibition and greater delay tolerance). Analysis will be conducted using regression approaches, as recommended by Valeri and Vanderweele in their 2013 paper [79] using SAS and SPSS; and path analysis using AMOS (for Windows) and Mplus (Muthen & Muthen, Los Angeles, CA.).

#### Moderator analysis

The presence of parental ADHD will be assessed using the CSS [65]. This score will be collected at baseline and an interaction term (CSS * treatment group) will be included in the mixed-effect regression models to determine whether parent training might be less beneficial for children whose parents demonstrate symptoms of ADHD. The interaction term will be considered significant for *P* < 0.05.

Risk of long-term burden as assessed by the PARI will be included in the mixed-effect regression models to determine whether this measure moderates the effect of treatment on primary and secondary outcomes.

#### Genetic moderation of parent-training effects

DNA will be collected from parents and children, to test whether parental responses to parent training in terms of changes in parenting behaviour and the parent’s responses to the child during interaction are moderated by functional polymorphisms in selected genes shown to affect parenting and ‘openness’. These will include polymorphisms that have been associated with the ADHD phenotype in genes that control serotonin, dopamine and oxytocin function (*DRD4*; *DRD2*; *5HTT-* group) will be included in the mixed-effect regression models to determine whether this measure moderates the effect of treatment on primary and secondary outcomes.

#### Effects as a function of treatment fidelity

Scores from the LOT and the Working Alliance Inventory will be used to calculate a fidelity composite measure (scored 1 to 3, indicating low to high fidelity). An interaction term ‘fidelity composite score * treatment group’ and, similarly, an interaction term ‘FSI * treatment group’ will be included in the mixed-effect regression models to determine whether these measures moderate the effect of treatment on primary and secondary outcomes.

### Health economic analysis

The economic analysis, adherent to guidelines for good economic evaluation practices [80], will adopt a societal perspective, including health and social services, as well as any costs borne by families. The primary economic evaluation will be in the form of cost-effectiveness analysis (CEA), and a secondary economic evaluation in the form of cost-utility analysis (CUA). These will be based on intention to treat and if approved, per-protocol analyses (see Statistical Analyses above).

#### Resource use and family borne costs

Detailed information on all resources required to provide both the adapted-NFPP and the IY programme will be collected. This will include the design of a time collection form to record therapist time spent providing the intervention inclusive of preparation and travel time. It will also include resources such as manuals and handouts, training and supervision and any necessary fidelity procedures. Resource use will be combined with relevant unit cost data to provide estimates of the costs of providing these interventions. The detailed breakdown of the types of costs required will allow carrying out sensitivity analysis of the effect of varying any aspect of how the interventions are provided. It will also allow comparison of the economics of different modes of provision.

Patient-reported resource-use measures are an important component and one of the most challenging parts of an economic evaluation. The CSRI [58] is considered one of few resource-use measures that have been comprehensively tested and the validity and reliability of the instrument is considered good, with consistency generally fairly well demonstrated [81]. An adapted version of the CSRI will be used to collect detailed information from all parents indicating contacts with frontline professionals or specialized services and will allow identification of ADHD related health and social care resource use required by individuals and families. This will include costs related to counselling services and support groups, child education and day care, as well as employment related costs due to child’s health/behaviour. There is no doubt that there is a tension between demand for information on costs and its supply; this comprehensive cost data collection will provide the opportunity to report on economic implications of ADHD in preschool children.

#### Primary outcome measure and health-related quality of life

The preference-based generic health-related quality-of-life (HRQoL) instruments enable comparisons across chronic conditions and benchmarking with healthy population samples. The economic evaluation alongside clinical trials framework also enables estimation of quality-adjusted life years (QALYs) and presentation of results in a cost per QALY context. There is some doubt as to how appropriate HRQoL scores will be in very young children. Given this, it is not yet possible to identify the best paediatric generic HRQoL instrument to be used in the preschool age group. There is a paucity of research on the use of preference-based measures of HRQoL such as the EQ-5D in very young children. However, there is evidence supporting the use of parent-proxy EQ-5D ratings of children with ADHD in the UK and US. Matza et al. [75] assessed the HRQoL of children with ADHD using the parents’ proxy version of the EQ-5D and reported that the instrument was able to detect impairment in children diagnosed with ADHD and that this measure was feasible and valid when used as part of an overall health outcomes assessment in clinical studies of childhood ADHD. Hence, the within-trial cost-effectiveness will be estimated using the EQ-5D to estimate QALYs (CUA).

Given the degree of change based on EQ-5D scores compared with the primary outcome measure (SNAP-IV-P), the effectiveness of this instrument in estimating HRQoL to be used in economic evaluations alongside clinical trials, for this age group, with ADHD symptoms, will also be investigated.

Following NICE recommendations [67], as part of the diagnostic process for children, there should also be an assessment of their parents’ or carers’ mental health; in this study, EQ-5D and EQ-5D VAS scores will be used to detect changes on HRQoL for both (a) the child, using proxy completion by the parent or main caregiver, and (b) the parent’s or main caregiver’s own state of health.

#### Cost-effectiveness analysis (CEA)

The aim of the CEA will be to determine whether the adapted-NFPP, a home-based parenting programme, is more cost-effective with children at high risk of poor outcomes than either a TAU group or a generic package (IY: active control).

In addition to cost per QALY (CUA), the within-trial CEA will also be estimated using the primary outcome measure SNAP-IV-P, looking at cost per unit change in the SNAP-IV-P score, to allow for the possibility that the EQ-5D is not sensitive to changes in ADHD symptoms.

The NICE [67] reviewed treatments for ADHD, and, in their economic evaluation, people with ADHD were either classified as responders or nonresponders, based on the Clinical Global Impression-Improvement (CGI-I) Scale. A study from Lloyd et al. [82] developed a classification system of four health states (normal; borderline to mildly ill; moderately to markedly ill and severely ill), reflecting different levels of severity of the patient’s symptoms. A similar approach will be explored, classifying children by severity using the primary outcome and the EQ-5D, depending on the variation in severity of those recruited. In addition, by mapping the severity scale to the improvement scale, the response to treatment will be estimated, to allow classification of children as responders or nonresponders, as described by NICE. Given that the adapted-NFPP has recently been adapted to address the specific needs of ‘hard-to-reach’ and ‘difficult-to-treat’ children, this analysis is appropriate. The results will be presented as point estimates and cost-effectiveness acceptability curves. Deterministic sensitivity analysis will be combined with probabilistic sensitivity analysis, to explore different types of uncertainty.

Missing data will be examined as to whether cases with missing data are similar to those with full economic data in a similar manner to the main trial analysis. If cases with missing data are similar to those with complete data, an analysis will be carried out using only cases with complete data (complete case analysis). If this is not the case, appropriate measures of imputing missing data, for example, multiple imputation, will be explored. This will be done in consultation with the trial statistician. If results using imputed values are produced, these will be in addition to those using a complete case analysis.

## Ethical approval

The study protocol was approved by the National Research Ethics Service, NRES Committee South Central for Portsmouth and the University of Southampton. The trial is registered with Current Controlled Trials (ISRCTN39288126).

## Discussion

The COPPI trial will provide vital information about the clinical value of parenting programmes for the treatment of ADHD in preschool children and will provide novel insights at a number of levels. For example, first, by contrasting a specialist intervention designed specifically to target core ADHD symptoms (adapted-NFPP) against a more generic parenting approach (IY), the trial is designed to answer questions about the specificity of effects of parenting programmes within this sample. Second, by adding a health economic component, the trial will be able to establish the value of any trade-off between the potentially greater costs of a home-based one-to-one intervention (adapted-NFPP) and a possibly less expensive group-based alternative. Third, by studying a sample enriched for hard-to-reach and difficult-to-treat families and preschool ADHD children, this trial will allow us to test whether programmes previously seen as effective with less challenging samples can also work with clinically more complex cases. Fourth, by taking direct observation measures along with parent and teacher ratings, it will allow us to disentangle the effects of rater (parent versus teachers versus observer) from those of setting (home versus school). Fifth, by testing for the mediating role of neuropsychological factors and altered parenting the trial will allow the examination of the processes that drive therapeutic change. Sixth, by collecting DNA and genotyping for ADHD risk and more general susceptibility genes, the study will allow us to isolate genetic factors that might determine clinical response and help to better target treatments in the future.

Attention-deficit/hyperactivity disorder is a major health burden across the life span. Effective and cost-effective treatments need to be developed and validated for use with a range of different sorts of children with ADHD. Early intervention, it has been argued, offers a number of advantages, especially in optimizing the value of nonpharmacological approaches. Establishing the value of the COPPI interventions will present a major step forward in the search for an effective early intervention strategy for ADHD.

## Trial status

COPPI is an ongoing trial and it is expected that participant recruitment will continue, subject to approval, until January 2014, when a final tranche of 12 week parenting programmes will commence.

## Appendix 1

### Adapted New Forest Parenting Programme (adapted-NFPP)

#### *The New Forest Parenting Programme (NFPP)*

This programme is centred around four broad themes:

1. The importance of psychoeducation; that the parent understands preschool-type ADHD problems, and therefore why the child may behave the way he or she does.

2. The parent–child interaction and how that might be enhanced.

3. Behavioural strategies that are targeted to the child and the child’s symptoms.

4. The neuropsychological deficits for children with ADHD, so work is done with the parent using attention training games (to work on auditory memory and visual memory) and helping the child cope with delay and waiting as well as self-organization.

Emphasis is also placed on scaffolding the parent to be the child’s trainer, so that, in turn, the parent can scaffold the child and work from the child’s level of development. The parent is encouraged to practise tasks outside the home and to use teachable moments to consolidate the learning. The importance of the therapist using material brought by the parents (diaries, discussion in sessions) to brainstorm strategies and the use of modelling and role play as techniques for the therapist is also emphasized. For some sessions, the child will also be present.

#### *Brief summary of the timetable for parent training*

The 12-week programme expands the time to deliver the programme from an 8-week programme, so that the programme can be delivered at a slower pace over the 12 weeks. To this programme, information has also been added about: children with sleep disorder; parents with a mental illness; parents with ADHD; parents with learning difficulties; children with learning difficulties or speech and language problems. The use of mindfulness and social stories has also been added. Further information has also been added in relation to motivational techniques and the use of social stories. Each week, the parent fills in a positive diary, a negative diary and, from Week 3, a play diary. The parent is given handouts after each session.

#### *Induction visit (child also present)*

The therapist delivering this new version will make an introductory visit to the family to introduce the programme and to assist in motivating the family. At this session, the therapist will explain how the programme will be delivered and arrange mutually convenient times for visits.

#### *Week 1*

##### Parent alone

Interview the parent to learn about this particular child’s problems and symptoms; to understand the child’s developmental history, to check for developmental delay, including speech and language issues; to learn more about the parents, their mental health, their level of education, and to understand any other factors that might be relevant to helping the parent and therapist work together.

Discuss characteristics of ADHD, acceptance of child, effectiveness of simple interventions. Emphasize importance of praise and the use of simple language. Introduce behaviour diary. The parent is given a diary to record positive and negative moments at home and how these were handled by the parent.

#### *Week 2*

##### Parent alone

Reinforce message from Week 1. Look at diary and discuss parent’s feelings about behaviour during week. Start discussing the effectiveness of simple interventions, recruiting attention, listening skills and eye contact. Emphasize importance of praise, of clear messages and short sentences, and the importance of clear boundaries.

Discuss practising techniques; teachable moments; attention training; impulsivity; the use of games. Give out diaries and a play diary to record how the play with games has gone.

#### *Week 3*

##### Parent alone

Reinforce messages from previous weeks. Examine diaries and discuss parent’s feelings. Introduce the concept of scaffolding. Discuss temper tantrums; emphasize firmness and voice control and keeping calm and focused; avoiding confrontation; the power of distraction; mirror images; the importance of routine and clear boundaries; countdowns. Play: introduce two new games.

#### *Week 4*

##### Parent and child

Reinforce messages from previous weeks and ensure that they have been implemented. Introduce concepts of ‘I’ messages; ‘we’ messages; enthusiastic tone of voice; speaking respectfully; use of choices and house rules. Play.

#### *Week 5*

##### Parent and child

Review Weeks 1 to 4, focusing on problems identified and solutions given. Assess parent’s ability to implement strategies. Review diaries, isolate examples, and discuss how parents cope; reinforce psychoeducation; discuss teachable moments; observing own child and planning ahead; attention training; distraction techniques; quiet time; time out; behaviour modification; speech and language development; parent–child relationship and mindfulness. Play.

#### *Week 6*

##### Parent and child

Consolidate the strategies and go over the ones with which the parents are having difficulty, using the diaries for material. Reinforce psychoeducation; attention training principles via play; teachable moments; speech and language development; behaviour modification; discussion and holding of parents, in order to explore parental mental health and emotions; stress positive interaction between mother and child by using video interaction techniques.

#### *Week 7*

##### Parent alone

Give feedback to the parent of the play session carried out in the previous week. Underline the importance of behavioural techniques discussed and learned and illustrate with examples from the previous weeks. Reinforce psychoeducation; holding of parents through change; stress positive interaction between mother and child by showing her the video; stress the importance of speech and language and development through play, through looking at books, nursery rhymes and social stories.

#### *Week 8*

##### Parent alone

Reinforce messages from previous weeks. Focus on one or two of the key areas of particular concern for each parent. Diaries should continue to be used to identify these key areas and to provide examples of good practice. Discuss how parent feels about continuing with the strategies. Reinforce psychoeducation; discussion and holding of parents in order to explore parental mental health and emotions; review the use of social stories; recap strategies. Review use of play.

#### *Week 9*

##### Parent and child

Reinforce psychoeducation; review the use of social stories; recap strategies; play session with parent and child.

#### *Week 10*

##### Parent and child

Reinforce psychoeducation; discussion about what is relevant to parent; review the use of social stories; recap strategies; play session with child.

#### *Week 11*

##### Parent alone

Reinforce psychoeducation; discussion about what is relevant to parent; review the use of social stories; recap strategies; positive feedback and praise to parent with viewing of previous week’s DVD. Endings: discussion that next week is last meeting.

#### *Week 12*

##### Parent alone

Reinforce psychoeducation; discussion about what is relevant to parent; review the use of social stories; recap strategies; positive feedback and praise to parent; endings; reinforce positive ways of problem-solving to cope with future difficult situations; discuss importance of continuing ‘playtime’ in preparation for school and friends; planning ahead (support network, encourage parents to keep diary to track progress) and praise the parents.

The parent will be reminded that the psychologist will be contacting the parent again within the next 2 weeks, to arrange a short visit to the parent’s home, at a time convenient to the parent, to carry out an interview for the purpose of completing assessments and administering questionnaires.

The parent will also be reminded during this visit that the psychologist will make contact again after 6 months has elapsed (from the date of completion of the parenting programme) to make arrangements for a similar short visit to the parent to carry out a further interview for the purpose of completing assessments and administering questionnaires. At the end of this visit, the research fellow will provide the parent with a debriefing letter.

## Appendix 2

### Incredible Years (IY) 12-week parenting programme

12 weekly sessions will cover the goals in the order outlined below.

#### *Child-directed play promotes positive relationships*

• Understanding the value of showing attention and appreciation as a way of increasing positive child behaviours;

• Understanding the importance of showing joy with the child through songs and games;

• Understanding how to promote imaginary and pretend play;

• Learning how to be child-directed and understanding its value for children;

• Learning how to end play successfully with the child;

• Learning about the child’s developmental needs and milestones;

• Learning about the ‘modelling’ principle;

• Balancing power between parent and child;

• Building the child’s self-esteem and creativity through child-directed play;

• Understanding the ‘attention rule’.

#### *Promoting language with child-directed coaching*

• Understanding how to model and prompt language development;

• Learning how to coach preschool readiness skills;

• Learning about ‘descriptive commenting’ and child-directed coaching;

• Learning about ‘persistence coaching’ to build the child’s ability to be focused and calm and to persist with an activity;

• Learning about the ‘modelling principle’;

• Understanding how to promote prereading and prewriting readiness skills;

• Appreciating normal differences in the child’s developmental abilities and temperament; completing temperament checklist.

#### *Social and emotion coaching*

• Understanding how to use emotion coaching to build the child’s emotional vocabulary and encourage self-expression of feelings;

• Understanding how to prompt social coaching to encourage the child’s social skills such as sharing, being respectful, waiting, asking, taking turns;

• Learning the ‘modelling principle’, by the parent avoiding the use of critical statements and demands and substituting positive polite language, the child learns more positive communication;

• Understanding how to coach sibling and peer play using modelling, prompting and praise to encourage social skills;

• Understanding developmental stages of play;

• Learning how to apply coaching principles in other settings such as mealtimes, bath time and grocery store trips.

#### *The art of praise and encouragement*

• Labelling praise ‘Give to get’ principle, for adults and children;

• Attending to learning ‘process’, not only end results;

• Modelling self-praise;

• Resistance to praise, the difficulties in giving and accepting praise;

• Promoting positive self-talk;

• Using specific versus nonspecific encouraging statements;

• Gaining and giving support through praise;

• Avoiding praising only perfection;

• Recognizing social and self-regulation skills that need praise;

• Building the child’s self-esteem through praise and encouragement.

#### *Spontaneous incentives for children*

• Shaping behaviours in the direction you want, ‘small steps’;

• Clearly identifying positive behaviour;

• Rewards are a temporary measure leading to the child learning a new behaviour;

• What will reinforce one child will not necessarily reinforce another;

• Value of unexpected and spontaneous rewards;

• Recognizing the ‘first-then’ principle;

• Designing programmes that are realistic and developmentally appropriate;

• Understanding how to set up programmes for problems such as not dressing, noncompliance, picky eating, difficulty going to bed, toilet training and rough animal care;

• Importance of reinforcing oneself, teachers, and others.

#### *Handling separations and reunions*

• Establishing clear and predictable routines for separating from the child;

• Establishing routines for greeting the child after being away from them;

• Understanding object and person permanence;

• Providing adequate monitoring at all times;

• Understanding how peek-a-boo games help children;

• Understanding how predictable routines for bedtime and schedules help the child feel secure and safe;

• Completing the child-proof home safety checklist.

#### *Positive discipline; effective limit setting*

• Reduce number of commands to only necessary commands;

• Learning about the importance of distractions and redirections;

• Understanding the value of giving the child some choice;

• Politeness principle and modelling respect;

• Clear and predictable household rules offer children safety and reduce misbehaviours;

• ‘Monitoring principle’: understanding the importance of constant monitoring and supervision for toddlers;

• All children will test rules, don’t take it personally;

• Commands should be clear, brief, respectful, and action-oriented;

• ‘When-then’ commands can be effective;

• Distractible children need warnings and reminders.

#### *Positive discipline; handling misbehaviour*

• Understanding how to use distractions and redirections coupled with ignore;

• Parents maintaining self-control using calm-down strategies and positive self-talk;

• Repeated learning trials, negative behaviour is a signal child needs some new learning;

• Using the ignore technique consistently and appropriately for selected behaviours, such as whining, tantrums;

• Knowing how to help children practice calming down;

• Know how to handle children who hit or bite;

• Understanding the importance of parents finding support.

## Abbreviations

ADHD: attention-deficit/hyperactivity disorder; adapted-NFPP: adapted New Forest Parenting Programme; CAMHS: child and adult mental health services; CEA: cost-effectiveness analysis; CGI-I: Clinical Global Impression-Improvement; CONSORT: Consolidated Standards of Reporting Trials; COPPI: Comparison of Preschool Parenting Interventions; CRF: case report form; CSRI-R: Revised Client Service Receipt Inventory, CSRI-R; CSS: Current Symptoms Scale; CUA: cost-utility analysis; DISC-IV: Diagnostic Interview Schedule for Children Version 4; EASI: Emotionality, Activity, Sociability and Impulsivity; ECBI: Eyberg Child Behaviour Inventory; EE: expressed emotion; EQ-5D-3 L: EuroQol 5 dimensions, 3 levels; FMSS: five-minute speech sample; FSI: Family Strain Index; GHQ: General Health Questionnaire; GIPCI: Global Impressions of Parent–child Interactions; HRQoL: health-related quality of life; IY: Incredible Years; LOT: Leader Observation Tool; MTA: Multimodal Treatment Study of Children with ADHD; NFPP: New Forest Parenting Programme; NICE: National Institute for Health and Care Excellence; NIHR: National Institute for Health Research; ODD: oppositional defiant disorder; PARI: Preschool ADHD Risk Index; PEDIA: Programme for Early Detection and Intervention for ADHD; PIP: Parent Involvement Project; QALY: quality-adjusted life year; RCT: randomized controlled trial; TAU: treatment as usual; UoSCTU: University of Southampton, Clinical Trials Unit; VAS: visual analogue scale; WAI-S: Working Alliance Inventory, Short Form; WWP: Werry-Weiss-Peters Activity Rating Scale.

## Competing interests

MT, CLB, ESB and DD are the original co-developers of the NFPP and benefit from royalties from the self-help version of the programme. MT and CLB run NFPP courses, for which they receive financial remuneration. In the last 3 years, ESB has received financial remuneration from Shire for consultancy, public speaking at educational events and advisory board membership and from Janssen Cilag and Medice for public speaking. He has received research funding from Shire. JH runs courses and supervision, for which she receives financial remuneration. JH is the UK representative of IY programmes that were developed by Carolyn Webster-Stratton, from whom the trial management group received approval for the use of the 12-week IY course in this trial.

## Authors’ contributions

ESB: conception and design, manuscript writing, critical revision and final approval of the manuscript. MT: conception and design, data acquisition and critical revision and final approval of manuscript. DCMcC: refinement of the trial design, acquisition of data, manuscript writing, critical revision and final approval of the manuscript. DD: refinement of the trial design, data collection, critical revision and final approval of the manuscript. JB and DC: refinement of the trial design, critical revision and final approval of the manuscript. JH and CLB: refinement of the trial design, data acquisition, critical revision of the manuscript and final approval of the manuscript. LS and LD: data analysis. TM: data analysis and critical revision of the manuscript. JC: data acquisition and analysis, drafting and final approval of the manuscript. JR and MC: economic data analysis, refinement of the trial design, critical revision and final approval of the manuscript. All authors read and approved the final manuscript.
